# The Influence of the Gut Microbiome on Host Metabolism Through the Regulation of Gut Hormone Release

**DOI:** 10.3389/fphys.2019.00428

**Published:** 2019-04-16

**Authors:** Alyce M. Martin, Emily W. Sun, Geraint B. Rogers, Damien J. Keating

**Affiliations:** ^1^Molecular and Cellular Physiology Laboratory, College of Medicine and Public Health, Flinders University, Adelaide, SA, Australia; ^2^Microbiome Research Laboratory, Flinders University, Adelaide, SA, Australia; ^3^Infection and Immunity, South Australian Health and Medical Research Institute, Adelaide, SA, Australia; ^4^Nutrition and Metabolism, South Australian Health and Medical Research Institute, Adelaide, SA, Australia

**Keywords:** enteroendocrine cells, microbiome, metabolism, GLP-1, PYY, GIP, serotonin, CCK

## Abstract

The microbial community of the gut conveys significant benefits to host physiology. A clear relationship has now been established between gut bacteria and host metabolism in which microbial-mediated gut hormone release plays an important role. Within the gut lumen, bacteria produce a number of metabolites and contain structural components that act as signaling molecules to a number of cell types within the mucosa. Enteroendocrine cells within the mucosal lining of the gut synthesize and secrete a number of hormones including CCK, PYY, GLP-1, GIP, and 5-HT, which have regulatory roles in key metabolic processes such as insulin sensitivity, glucose tolerance, fat storage, and appetite. Release of these hormones can be influenced by the presence of bacteria and their metabolites within the gut and as such, microbial-mediated gut hormone release is an important component of microbial regulation of host metabolism. Dietary or pharmacological interventions which alter the gut microbiome therefore pose as potential therapeutics for the treatment of human metabolic disorders. This review aims to describe the complex interaction between intestinal microbiota and their metabolites and gut enteroendocrine cells, and highlight how the gut microbiome can influence host metabolism through the regulation of gut hormone release.

## Introduction

The gastrointestinal (GI) tract is host to a highly complex microbial ecosystem, comprising of bacteria, yeast, fungi, bacteriophages, and other viruses (Scarpellini et al., 2015), as well as protozoa and archaea (Koskinen et al., 2017; Laforest-Lapointe and Arrieta, 2018). Commensal bacteria, hereto referred to as gut microbiota, are found along the length of the GI tract and at greatest density within the caecum and colon, and along with their genes and gene products (collectively referred to as the gut microbiome), perform several functions that heavily influence host physiology. Not only does the gut microbiota play a critical role in modulating host immune defense (Belkaid and Hand, 2014) and brain function (Rogers et al., 2016), it also plays a role in regulating host metabolism (Turnbaugh et al., 2006; Turnbaugh and Gordon, 2009; Vrieze et al., 2012, 2014; Le Chatelier et al., 2013; Nieuwdorp et al., 2014; Blaut, 2015; Hartstra et al., 2015; Seeley et al., 2015; Suarez-Zamorano et al., 2015; Aguirre et al., 2016; Pedersen et al., 2016; Molinaro et al., 2017; Rodrigues et al., 2017; Brubaker, 2018; Fabbiano et al., 2018). This is well-illustrated by the transfer of microbiota from lean and obese human twins into germ-free (GF) mice lacking a native gut microbiome, resulting in the conveyance of the metabolic phenotype of the host (Ridaura et al., 2013). Microbiota depletion in mice confers significant protection against metabolic dysregulation induced by a high-fat diet such as obesity, glucose intolerance and insulin resistance (Suarez-Zamorano et al., 2015), all of which are hallmarks of metabolic diseases including type 2 diabetes (T2D). While bacteria-mediated inflammation is associated with detrimental metabolic effects in mice (Lam et al., 2012; Molinaro et al., 2017), the underlying mechanisms by which gut microbiota influence metabolism are still not fully understood. The gut microbiome contributes significantly to host energy harvest by converting inaccessible nutrient sources such as plant polysaccharides and other complex carbohydrates, into readily absorbable metabolites (Tremaroli and Backhed, 2012). Moreover, a key link has been established between the gut microbiome and the release of several gut hormones that are important regulators of peripheral metabolism.

**FIGURE 1:**
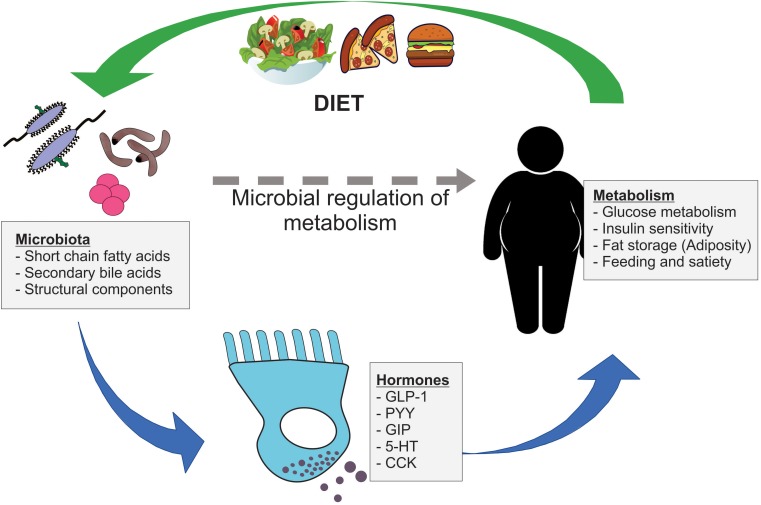
Microbial regulation of host metabolism via gut hormone release. Gut microbiota signal to nearby enteroendocrine (EE) cells via a range of microbial metabolites, including short chain fatty acids and secondary bile acids, and structural components. These EE cells release important metabolically active hormones, such as GLP-1, PYY, GIP, 5-HT, and CCK, which influence key metabolic processes including glucose metabolism, insulin sensitivity, adiposity, and feeding behavior. In turn, dietary components impact the composition of gut microbiota, which may have further downstream consequences on gut hormone secretion and host metabolism.

Within the mucosal lining of the gut, specialized enteroendocrine (EE) cells synthesize and secrete several hormones that facilitate a range of key physiological processes. Collectively, EE cells constitute the largest endocrine organ in the body (Ahlman and Nilsson, 2001), despite making up less than 1% of the total epithelial cell population in the gut. A broad number of EE cell subpopulations have been defined, based largely on their hormone expression profile (Fothergill et al., 2017). EE cells have the capacity to sense the luminal nutrient environment of the gut and are differentially responsive to many dietary compounds and luminal conditions within the intestine. Mounting evidence has highlighted that the gut microbiome influences EE cell hormone release, with downstream consequences for host metabolism and metabolic disease progression (Figure 1). There is also recent evidence that microbial-mediated release of gut hormones may influence other EE cell types (Lund et al., 2018), demonstrating the complexity that is the relationship between the gut microbiome, gut hormone release, and host metabolism. This review aims to describe how intestinal microbiota and their metabolites can influence host metabolism through the regulation of gut hormone release.

## Microbial Metabolites Signal With Host Cells

### Short Chain Fatty Acids

**FIGURE 2:**
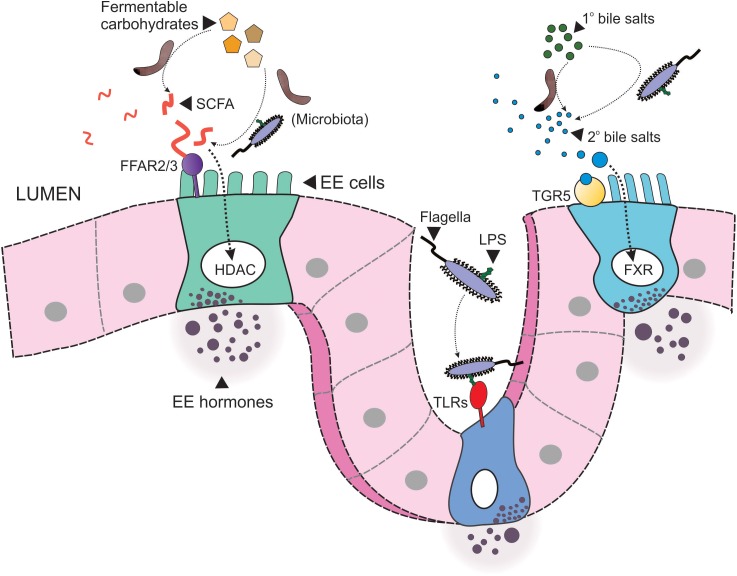
Microbial signaling to enteroendocrine cells. Resident microbiota within the intestinal lumen signal to enteroendocrine (EE) cells via multiple pathways. Firstly, microbiota convert indigestible carbohydrates to short chain fatty acids (SCFA), which in turn signal to EE cells via free fatty acid receptors 2 or 3 (FFAR2/3) or by activation of nuclear histone deacetylases (HDAC). Secondly, microbiota convert primary (1°) bile acids to secondary (2°) bile acids, such as deoxycholate, which then signal to EE cells via the membrane G protein-coupled bile acid receptor, TGR5, or nuclear FXR. Finally, structural components of microbiota, such as flagella, and the endotoxin lipopolysaccharide (LPS), signal to toll-like receptors (TLRs).

The gut microbiota produces an array of metabolites through the breakdown of indigestible carbohydrates (Figure 2). The most abundant of these metabolites are the short chain fatty acids (SCFAs) acetate, propionate and butyrate (Topping and Clifton, 2001), which exist at a ratio of approximately 3:1:1 in the human intestinal lumen, respectively (Cummings et al., 1987; Mowat and Agace, 2014), however, this ratio is, at least in part, dependent upon both diet and microbial composition. The fate of these bacteria-derived SCFAs differs substantially: acetate is readily absorbed into the circulation for distribution to peripheral tissues. Propionate, on the hand, is metabolized by the liver upon absorption (Koh et al., 2016), while the majority of butyrate is consumed locally by colonocytes as their primary fuel source. While the majority of bacteria-derived SCFAs are present in the colon, lesser amounts have also been detected in the ileum of pigs, as a result of cecoileal reflux (Cuche and Malbert, 1999), and to a lesser extent, the proximal small intestine. The relative abundance of SCFA is also likely to differ along the length of the gut as a result of the region-specific microbial composition, substrate exposure, and absorption (Gu et al., 2013). For example, genes encoding carbohydrate metabolism pathways are enriched in members of the Bacteroidetes phylum, while genes encoding bile acid metabolism pathways are enriched in the bile acid-tolerance Firmicutes (David et al., 2014).

Receptors present on EE cells allow these cells to sense luminal and possibly circulating SCFA, which triggers the release of several metabolically active gut hormones. SCFA signal via two predominant mechanisms: (1) inhibition of nuclear histone deacetylase (HDAC) (Waldecker et al., 2008; Fellows et al., 2018; Larraufie et al., 2018) to alter gene transcription and expression, and (2) stimulation of G-protein-coupled free fatty acid receptors 2 and 3 (FFAR3, FFAR3), expressed throughout the length of the GI tract in distinct regional patterns. FFAR2 has equal affinity for acetate, propionate and butyrate, while the affinity of FFAR3 for acetate is substantially lower than for propionate and butyrate (Offermanns, 2014). Signaling of SCFA via FFAR2/3 is therefore dependent upon the combination of receptor type and specific metabolite abundance.

### Secondary Bile Acids

Bile acids are amphipathic molecules synthesized by hepatocytes from cholesterol and are released into the GI lumen to aid the solubilization, and thus absorption, of dietary lipids. It has long been appreciated that the intestinal microbiota is directly involved in host bile acid metabolism, as effective enterohepatic recycling of bile salts is heavily reliant on deconjugation and dihydroxylation of bile acids by microbial-derived bile salt hydrolases (BSH). This gives rise to secondary bile acids (Jones et al., 2008), which are more hydrophobic and can thus be reabsorbed via passive diffusion, limiting bile acid loss through feces. Specific activity of BSH in human gut microbiota differs between different phyla, with BSH in the Firmicutes and Actinobacteria capable of metabolizing all conjugated bile salts and Bacteroidetes BSH activity being specific to tauro-conjugated bile acids (Jones et al., 2008). In addition to this role in the GI tract, bile acids are signaling molecules that are implicated in peripheral metabolism. Bile acids have major roles in peripheral metabolism through their action on two bile acid receptors, the G-protein coupled receptor TGR5 (formerly known as Gpbar1), and the nuclear receptor FXR, both of which are expressed in EE cells (Figure 2). Receptor affinity and potency varies substantially between different bile acids. As such, the gut microbiome can exert profound influence on host metabolism by altering the composition of the bile acid pool, through altered bile acid synthesis and re-uptake.

### Cellular Recognition of Microbial Structural Components

Structural components of the microbial membrane, such as flagella and membrane-bound lipopolysaccharide (LPS), act as signaling molecules through a number of cellular pattern recognition proteins (Gordon, 2002). LPS is a cell-wall component of Gram-negative bacteria, such as members of the Bacteroidetes phylum, and is a potent ligand for toll-like receptors (TLRs), particularly toll-like receptor 4 (TLR4). In addition to powerful immunity- and inflammation-inducing effects (Takeuchi and Akira, 2002; Lancaster et al., 2018), the expression of TLRs has been demonstrated in a number of EE cells and activation of this receptor triggers secretion of a number of metabolically active hormones such as GLP-1 (Lebrun et al., 2017), 5-HT (Kidd et al., 2009), and PYY (Larraufie et al., 2017; Figure 2). Clinically, elevated levels of LPS (endotoxemia) are closely associated with obesity and insulin resistance (Cani et al., 2007a). Mechanisms by which LPS contribute to perturbed glycemic control and adiposity likely involve complex interactions between gut hormone secretion, mucosal barrier integrity, and host inflammation and immune pathways.

## The Microbiome Regulates Host Metabolism Via Gut Hormone Release

### Serotonin

Enterochromaffin (EC) cells are the source of almost all (about 95%) serotonin (5-HT; 5-hydroxytryptamine) within the body. These cells constitute almost half of all EE cells and are dispersed throughout the length of the GI tract in varying densities (Raghupathi et al., 2013). EC cells have long been known to be important in many intrinsic gut mechanisms associated with motility (Keating and Spencer, 2010; Spencer et al., 2011, 2015; Spencer and Keating, 2016; Keating and Spencer, 2018) and EC cells are able to sense their local nutrient environment and respond by secreting 5-HT in a unique manner (Zelkas et al., 2015; Raghupathi et al., 2016; Thorn et al., 2016). There is now firm evidence that gut-derived 5-HT is a key driver for dysregulation of peripheral metabolism (Sumara et al., 2012; Watanabe et al., 2014; Crane et al., 2015; Young et al., 2015, 2018; Martin et al., 2017c). The absence of gut-derived 5-HT, through pharmacological inhibition or genetic ablation of the rate-limiting enzyme for 5-HT synthesis in the gut, tryptophan hydroxylase 1 (TPH1), conveys protection from diet-induced obesity in mice (Crane et al., 2015). Moreover, circulating 5-HT is increased in obese humans and is positively correlated with body mass index (Young et al., 2018) and poor glycaemic control (Takahashi et al., 2002).

The gut microbiome influences 5-HT levels in the host. GF and antibiotic-treated mice have substantially lower levels of EC cell-derived 5-HT when compared to conventionally raised (CONV-R) controls, which are restored by colonization of GF mice with donor gut microbiota (Yano et al., 2015). EC cells have the capacity to sense microbial metabolites, as they express both FFAR2 and FFAR3 (Akiba et al., 2015; Martin et al., 2017b), and a number of olfactory receptors (Bellono et al., 2017; Lund et al., 2018). Acute exposure of mouse primary EC cells to SCFA in culture does not, however, elicit an increase in 5-HT secretion (Martin et al., 2017a). Rather, the increase in 5-HT observed in the presence of a gut microbiome (Reigstad et al., 2015; Yano et al., 2015) is likely due to the chronic exposure mediating an increase in the biosynthesis of 5-HT, contributed to by increased EC cell proliferation (Yano et al., 2015). In addition, luminal butyrate infusion restores intestinal motility in GF mice, which is blunted in TPH1-KO mice, indicating the effects of butyrate may be mediated by EC cell 5-HT (Vincent et al., 2018). Acute responses to aromatic metabolites, such as isobutyrate and isovalerate, have been observed in EC cells within intestinal organoid preparations (Bellono et al., 2017), likely via olfactory receptor activation. However, it is plausible that this is an indirect response, due to the cross-talk with other gut-derived hormones such as GLP-1, which are also increased following exposure to microbial metabolites and have the capacity to signal to EC cells (Lund et al., 2018).

### GLP-1

Glucagon-like peptide 1 (GLP-1), a cleavage product of proglucagon, is secreted by L-cells predominantly located in the ileum and colon. GLP-1 is an incretin hormone (Kreymann et al., 1987), released postprandially and in response to nutrients such as glucose (Sun et al., 2017) to augment insulin and inhibit glucagon secretion from the pancreas (Grondahl et al., 2017). In addition, GLP-1 inhibits gastric emptying and influences satiety and food intake (Holst, 2007). Together with PYY, GLP-1 is thought to underlie some of the metabolic gains observed following gastric bypass surgery (Madsbad and Holst, 2014) and the action of GLP-1 underlies some of the glucose-lowering ability of the diabetes therapy, metformin (Bahne et al., 2018). As such, GLP-1-targeted therapeutics including GLP-1 analogs and inhibitors of GLP-1 degradation by the enzyme dipeptidyl peptidase IV (DPP-4) have been extensively exploited for their anti-diabetic properties (Aroda et al., 2012). Interestingly, the microbiota possess DPP-4-like activity (Olivares et al., 2018b), which in mice is reduced by administration of the DPP-4 inhibitor, vildagliptin, and is accompanied by a shift in microbial composition that is independent of the direct effects of DPP-4 inhibition on microbiota function (Olivares et al., 2018a). Specifically, vildagliptin treatment is associated with a decrease in the abundance of *Oscillibacter* and increased in the abundance of *Lactobacillus*, with a reduction in TLR ligands and an increase in propionate (Olivares et al., 2018a). Thus, the DPP-4-like activity of intestinal bacteria can potentially influence the levels of circulating GLP-1 and PYY, which in turn may exist as a feedback loop to influence microbial composition and microbial metabolite abundance.

A dynamic relationship exists between L-cells and the gut microbiome. Bile acid-mediated activation of TGR5 (Bala et al., 2014), SCFA signaling (Tolhurst et al., 2012), LPS, and other metabolites such as indole (Chimerel et al., 2014) are all potent GLP-1 secretagogues. The regulation of GLP-1 by secondary bile acids is dependent upon the receptor-signaling pathway involved, as activation of TGR5 increases GLP-1 secretion while, on the other hand, activation of FXR reduces GLP-1 secretion. This dynamic relationship is made even more complex by TGR5-FXR cross-talk that exists between these two receptors, particularly in the colon (Pathak et al., 2017). Luminal infusion of the bile acid chenodeoxycholic acid, in rats, triggers release of GLP-1 into the vasculature, in addition to PYY, via TGR5 (Kuhre et al., 2018). Indole, another major bacterial metabolite derived from dietary tryptophan, acutely stimulates GLP-1 by prolonging cellular action potential duration (Chimerel et al., 2014). Conversely, chronic exposure to indole dose-dependently decreases GLP-1 secretion in primary murine L cells by inhibiting ATP synthesis pathway (Chimerel et al., 2014). In addition, microbial LPS triggers GLP-1 secretion via TLR4 following mucosal barrier injury (Lebrun et al., 2017), and as such, glucose-stimulated insulin secretion in a mouse model of endotoxemia (Nguyen et al., 2014). Dietary prebiotics such as oligofructose, which increase bacterial SCFA production, are associated with upregulated L-cell differentiation and GLP-1 content in the rat proximal colon, and reduces weight gain when administered before and during a HFD (Cani et al., 2007b). Increasing L-cell numbers, whereby increasing post-prandial GLP-1 release, are also associated with enhanced satiety and reduced adiposity (Cani et al., 2007b). The mechanisms by which SCFA increase GLP-1 secretion are region-dependent, as signaling in the small intestine is predominantly via FFAR3, whereas FFAR2-mediated GLP-1 release occurs in the colon (Greiner and Backhed, 2016).

Paradoxically, GF and antibiotic-treated mice have higher circulating GLP-1 levels during fasting (Zarrinpar et al., 2018) and reduced mucosal GLP-1 content (Duca et al., 2012), compared to genetically identical CONV-R mice. A recent study (Arora et al., 2018) reported that the gene expression profile of ileal L-cells derived from GF mice differed substantially from CONV-R mice. Notably, many of the genes regulating L-cell functional capacity are upregulated in GF mice and L-cells have a greater number of secretory vesicles in GF mice. What underlies these differences in GF mice is unknown but may be a reflection of GLP-1 resistance, which is observed in diet-induced obesity and associated with altered microbiota composition, particularly in the ileum (Grasset et al., 2017).

### PYY

Peptide tyrosine-tyrosine (PYY) is synthesized and secreted by L-cells, in addition to GLP-1, and is predominantly expressed in the lower small intestine and colon. PYY regulates food intake and satiety through activation of central G protein-coupled Y2 receptors on neuropeptide Y (NPY) and AgRP neurons in the hypothalamic arcuate nucleus (Dumont et al., 1995). This initiates a signaling cascade whereby appetite-stimulating NPY neurons are suppressed, allowing for the disinhibition of the satiety-inducing proopiomelanocortin (POMC)/α-MSH pathway (Loh et al., 2015). Obese humans have reduced circulating PYY (Batterham et al., 2006), as a result of attenuated colonic PYY secretion (le Roux et al., 2006), rather than PYY-resistance (Batterham et al., 2003). Circulating PYY exists as two forms: PYY_1-36_ and the DPP-4-cleaved PYY_3-36_, with the latter being the most dominant postprandial circulating form (Grandt et al., 1994) and the most biologically potent with respect to its anorectic effects (Chelikani et al., 2006).

The ability of gut microbiota to influence PYY secretion therefore has significant implications for the development of obesity and metabolic disease. Microbial SCFAs, particularly butyrate, cause a dose- and time-dependent increase in *PYY* gene expression in two EE model cell lines and in primary human colonic cell cultures (Larraufie et al., 2018). In addition, oral administration of butyrate moderately increases circulating PYY (Lin et al., 2012). The mechanisms by which SCFA increase the biosynthesis of PYY appear to be via a combination of FFAR2/3 signaling by all SCFA, and inhibition of HCAD by propionate and butyrate (Larraufie et al., 2018). Although, these mechanisms appear to be species-specific (Larraufie et al., 2018) and were not accompanied by an increase in GLP-1 secretion that is seen following exposure of primary mouse colonic cultures to propionate (Psichas et al., 2015). The use of a FFAR2 knockout mouse demonstrates the involvement of SCFA signaling in increasing the number of PYY-containing cells, particularly in mice exposed to a diet rich in the SCFA-precursor, inulin (Brooks et al., 2017). Alteration of the human gut microbiota through a 4-day broad-spectrum antibiotic regimen, acutely and reversibly increased postprandial plasma PYY (Mikkelsen et al., 2015). However, the precise alterations in microbial metabolites and bacterial species that underlie this change are unknown. Secondary bile acids are also potent stimuli for PYY secretion and the mechanisms by which this occurs are consistent with those for GLP-1 secretion (Kuhre et al., 2018). Luminal perfusion of a mixture of both primary and secondary bile acids into a vascularly perfused rat lumen increases venous effluent PYY levels in a TGR5-dependent manner, while the same effect was observed with infusion of the secondary bile acid CDCA alone (Kuhre et al., 2018).

### GIP

Glucose-dependent insulinotropic peptide (GIP), also known as gastric inhibitory peptide, is an incretin hormone released postprandially in the small intestine from classically defined K cells (Buffa et al., 1975). The activity of GIP is conveyed through GIP receptors (GIPR) expressed in pancreatic β-cells (Gremlich et al., 1995), adipocytes (Yip et al., 1998), bone cells (Bollag et al., 2000), and in neurons of the CNS (Paratore et al., 2011). Similar to GLP-1, the biological activity of GIP is rapidly attenuated by enzymatic breakdown by DPP-IV (Baggio and Drucker, 2007). Within the pancreas, GIP contributes significantly to postprandial insulin secretion, through increased insulin biosynthesis (Baggio and Drucker, 2007) and upregulated β-cell proliferation (Widenmaier et al., 2009). Defective GIP-signaling is believed to underlie, at least in part, the attenuated glucose-stimulated insulin secretion seen in T2D individuals (Vilsboll et al., 2002). GIP is also widely considered an adipogenic hormone (Thondam et al., 2017) as it promotes lipid uptake and storage in adipocytes (Getty-Kaushik et al., 2006).

Elevated GIP level was associated with the observed increased adiposity induced by a sub-therapeutic antibiotic regimen administered to mice at weaning for 7-weeks, as the treatment did not alter plasma levels of other gut hormones (Cho et al., 2012). The treatment significantly increased Firmicutes/Bacteroidetes ratio and caecal SCFA levels, which could potentially underlie the increased GIP level and thus, the increased adiposity. However, recent contradictory evidence demonstrates that carbohydrates within the lumen inhibit GIP secretion, via the microbial SCFA-FFAR3 signaling pathway (Lee et al., 2018). Whether the discrepancy seen across these studies is due to differential signaling via FFAR2 and FFAR3 is unknown. Consistent with specific receptor pathways for SCFA-mediated gut hormone release, oral administration of sodium butyrate into mice has been shown to transiently increase GIP and GLP-1 secretion, while sodium pyruvate and a SCFA cocktail are selective for increased GIP, but not GLP-1 or PYY (Lin et al., 2012).

### CCK

Cholecystokinin (CCK) is derived from the classically named “I cells” predominantly localized to the upper small intestine (Dockray, 2012). CCK is released in response to dietary fat and protein intake. CCK has well-defined roles in appetite regulation (Ritter, 2004; Becskei et al., 2007), gastric emptying and motility (Raybould and Tache, 1988; Raybould, 1991; Ellis et al., 2013) and the release of bile acids and pancreatic enzymes that are important for digestion (Li and Owyang, 1994; Li and Owyang, 1996; Owyang and Logsdon, 2004), through activation of CCK receptors (Rogers and Hermann, 2008). Less is known about gut microbial regulation of CCK compared to other gut hormones, largely due to the exposure of CCK-containing cells to microbiota limited to the small intestine. In pigs, ileal infusion of the SCFAs acetate, propionate and butyrate during feeding increased plasma CCK levels and paradoxically inhibits pancreatic secretion (Sileikiene et al., 2008). Limited investigations have been undertaken into microbial regulation of CCK in humans, however. One report from Roux-en-Y gastric bypass patients has revealed no changes in circulating CCK levels across normal weight and obese individuals pre- and post-surgery, despite a significant shift in microbial composition following surgery (Federico et al., 2016). However, reduced CCK protein expression is observed in dissociated cells from the proximal small intestine of GF mice, which was not due to reduced numbers of EE cells (Duca et al., 2012).

### Diet Influences Gut Microbiota Composition

The diversity of gut microbiota and relative abundance of microbial metabolites (metabolomic profile) is heavily dependent on specific dietary components (David et al., 2014), as evidenced by the substantial difference in microbial communities with consumption of plant-rich or protein-rich diets. This is due to the nutrient-induced selective pressures placed on microbiota, favoring bacterial species enrichened in the genes required for specific substrate metabolism. For example, plant-based diets and intake of probiotics increases luminal fiber and complex carbohydrate content, whereby selecting for species enriched in carbohydrate-active enzymes (David et al., 2014). Animal-based diets rich in fats and proteins and low in fiber increase luminal bile acid content, favoring bile acid-resistant microbes enriched with genes for bile acid metabolism, such as bile acid hydrosases and sulfite reductase (Devkota et al., 2012; David et al., 2014). Dietary fiber is also a major influence on gut transit (reviewed in detail by Muller et al., 2018), which, in turn, is an important determinant of fecal microbiome composition and metabolism (Roager et al., 2016; Vandeputte et al., 2016). In the absence of dietary fiber, however, a compensatory shift in the gut microbiome has been observed, with an increase in populations expressing mucin-degrading enzymes, suggesting an overall microbial preference for fiber-based substrates (Desai et al., 2016).

The increased consumption of non-nutritive sweeteners (NNS), such as saccharin, sucralose and aspartame, while being acutely beneficial for reducing caloric intake and blood glucose excursions, also has long-term consequences for microbiome composition and glucose intolerance. Notably, consumption of common NNS has been demonstrated in mice to exacerbate the development of glucose intolerance (Suez et al., 2014, 2015) and weight gain in males (Bian et al., 2017), which is mediated by distinct functional alterations to the gut microbiome (Suez et al., 2014). Specifically, the NNS saccharin and acesulfame-K increased the abundance of members of Bacteroidetes (Suez et al., 2014; Bian et al., 2017) and reduced abundance of Firmicutes (Suez et al., 2014). The effects of NNS on metabolism and microbial composition in humans is largely dictated by the native microbial composition prior to NNS exposure (Suez et al., 2014), while in mice, the response also appears to be gender-specific (Bian et al., 2017). Nevertheless, the NNS-induced changes in microbial composition observed in these studies is consistent with the microbial composition seen with obesity and metabolic disease (Ley et al., 2006; Turnbaugh and Gordon, 2009; Turnbaugh et al., 2009).

The richness and diversity of the human gut microbiome correlates with metabolic function (Le Chatelier et al., 2013; David et al., 2014; Dao et al., 2016) and a core obesogenic microbiome has been established, characterized by a high ratio of Firmicutes to Bacteroidetes (Ley et al., 2006; Turnbaugh and Gordon, 2009; Turnbaugh et al., 2009). Shifting the composition of the microbiome to reduce the abundance of Bacteroidetes, as seen with short-term caloric restriction, conveys improved metabolic outcomes in mice as a result of decreased LPS production and reduced TLR4 signaling (Fabbiano et al., 2018). There are also large-scale observational studies that linked antibiotic use to risk of type 2 diabetes (Boursi et al., 2015; Mikkelsen et al., 2015), although caution should be excised in interpreting these results, as hyperglycaemia is a risk factor for infection (Falagas and Kompoti, 2006) and may thus warrant increased antibiotic use. In addition, specific clinically used antibiotics, particularly vancomycin-imipenem and ciprofloxacin, induce differential effects on microbiome composition and microbial metabolite abundance following regrowth (Choo et al., 2017), which may have downstream implications for antibiotic-specific effects on metabolism. Ingestion of xenobiotics, such as pharmaceuticals and environmental chemicals, has the potential to modify gut microbial composition with downstream consequences for metabolism. This is evidenced by the diabetes drug, metformin, for which a shift in gut microbial composition is in part responsible for its therapeutic effects (Wu et al., 2017). The metabolism of xenobiotics by gut microbiota is also chemically distinct (Koppel et al., 2017), which highlights the gut microbiome as a possible tool for targeted drug design and delivery. Recent work by Vangay et al. (2018) has elegantly shown that migration from a non-Westernized culture to a Westernized culture rapidly and inter-generationally impacts the diversity of gut microbiota. This loss of microbial complexity and biodiversity resulted in a loss of key microbial enzymes required for plant fiber digestion, partly attributed to altered dietary composition and reduced food diversity, and may predispose individuals to metabolic disease (Vangay et al., 2018). As such, interventions to shift gut microbiota composition may be a powerful therapeutic tool for the treatment of obesity and metabolic disorders.

## Author Contributions

All authors listed have made a substantial, direct and intellectual contribution to the work, and approved it for publication.

## Conflict of Interest Statement

The authors declare that the research was conducted in the absence of any commercial or financial relationships that could be construed as a potential conflict of interest. The handling Editor declared a past collaboration with one of the authors DK.
